# Membrane of Functionalized Reduced Graphene Oxide Nanoplates with Angstrom-Level Channels

**DOI:** 10.1038/srep28052

**Published:** 2016-06-16

**Authors:** Byeongho Lee, Kunzhou Li, Hong Sik Yoon, Jeyong Yoon, Yeongbong Mok, Yan Lee, Hong H. Lee, Yong Hyup Kim

**Affiliations:** 1 BK21 Plus Program in Chemical Engineering, Seoul National University, Daehak-dong, Gwanak-gu, Seoul 151-742, Republic of Korea; 2 School of Mechanical and Aerospace Engineering, Seoul National University, Daehak-dong, Gwanak-gu, Seoul 151-742, Republic of Korea; 3 School of Chemical and Biological Engineering, College of Engineering, Institute of Chemical Processes(ICP), Seoul National University(SNU), Gwanak-gu, Daehak-dong, Seoul 151-742, Republic of Korea; 4 Asian Institute for Energy, Environment & Sustainability (AIEES), Seoul National University (SNU), Gwanak-gu, Daehak-dong, Seoul 151-742, Republic of Korea; 5 Department of Chemistry, Seoul National University, Daehak-dong, Gwanak-gu, Seoul 151-747, Republic of Korea

## Abstract

Membranes with atomic level pores or constrictions are valuable for separation and catalysis. We report a graphene-based membrane with an interlayer spacing of 3.7 angstrom (Å). When graphene oxide nanoplates are functionalized and then reduced, the laminated reduced graphene oxide (rGO) nanoplates or functionalized rGO membrane is little affected by an intercalated fluid, and the interlayer spacing of 3.7 Å increases only to 4.4 Å in wetted state, in contrast to the graphene oxide (GO) membrane whose interlayer spacing increases from 9 Å to 13 Å in wetted state. When applied to ion separation, this membrane reduced the permeation rate of small ions such as K^+^ and Na^+^ by three orders of magnitude compared to the GO membrane.

Membranes with angstrom-level pores or constrictions are of particular interest for water purification including desalination^1^, for separation of organic mixtures^2^ and hydrogen^3^, and for genetic and medical diagnostics^4^. Confinement in such pores or constrictions can even lead to a change in the state of a matter as reported recently on the formation of square ice in a 3.4 angstrom wide slit^5^. It is of interest that atomically thin porous graphene was also utilized for high permeance^6^. Graphene oxide nanoplates have received much attention in the fields of environment and energy^7^,^8^,^9^,^10^.

With the advent of laminates of graphene oxide (GO) nanoplates as membranes or GO membranes^11^,^12^, membranes of angstrom-range constrictions have become readily accessible and useful because of easy fabrication of the membrane and its robust nature in terms of mechanical strength and flexibility even for submicron-thick films. The finding that water can readily permeate through these films^13^ has made them more attractive as a membrane, particularly for ion sieving. However, the GO membranes have been found to be rather ineffective for sieving small ions such as potassium and sodium^14^,^15^,^16^,^17^. This low rejection rate of small ions has been attributed to an increase in the interlayer spacing from 9 Å to 13 Å when wetted^18^. Here we present a membrane based on GO with the interlayer spacing of 3.7 Å that is little affected by intercalated water and demonstrate the utility of the membrane for ion sieving.

The relatively high interlayer spacing of GO laminates is mainly due to the presence of functional groups on the basal plane of the nanoplate and on its periphery. Removal of these functional groups by reduction would certainly result in a significant decrease in the interlayer spacing. However, the reduction could make the membrane impermeable to water. In fact, the membrane of laminated reduced graphene oxide (rGO) nanoplates or rGO membrane is known to be practically impermeable to water^19^. One way of reducing GO and yet retaining the permeability is to functionalize GO with a species that will provide hydrophilicity. This functionalized GO can then be reduced for the purpose of decreasing the interlayer spacing, thereby reducing the spacing while retaining water permeability.

## EDTA Functionalized Graphenes

This functionalized rGO nanoplates were prepared following the method given by Hou *et al*.^20^. In this method, GO nanoplates are functionalized with N-(trimethoxysilylpropyl) ethylenediamine triacetic acid (EDTA-silane). This GO functionalized by the silylation (EDTA-GO) is then reduced with hydrazine to prepare the functionalized rGO nanoplates (EDTA-rGO). These plates of EDTA-GO and EDTA-rGO were dispersed in water (Supplementary Fig. 1), filtered, and then vacuum dried on polytetrafluoroethylene (PTFE) membrane, which was used as a support layer (pore size: 450 nm), to prepare EDTA-GO and EDTA-rGO membrane, respectively (Supplementary Fig. 2). Membranes of laminated GO and rGO nanoplates were also prepared for comparison. One interesting point is that EDTA-rGO is electrically conductive due to the removal of O-containing functional groups although the EDTA-GO is non-conductive (Supplementary Fig. 3).

The FTIR (Fourier Transform Infrared spectroscopy) spectra of the GO, EDTA-GO and EDTA-rGO are shown in Supplementary Fig. 4. Several new peaks can be observed in the spectra of EDTA-GO and EDTA-rGO when compared with those of GO, which indicates the presence of EDTA chain on the sheet surface. The two new bands at approximately 2900 and 2800 cm^−1^ in the spectra of EDTA-GO and EDTA-rGO are associated with the stretching of the methylene groups of the EDTA molecules^20^. The band at 1045 cm^−1^ in EDTA-GO spectrum and the band at 1118 cm^−1^ in EDTA-rGO spectrum were assigned to the formation of Si-O-C^20^,^21^. Sodium and Si backbone from EDTA molecule on the graphene is verified from EDS (energy dispersive spectroscopy, Supplementary Fig. S5) spectra. From the FTIR and EDS data, we conclude that GO was functionalized with EDTA molecules and EDTA-GO was reduced without loss of EDTA molecules when reduced by hydrazine.

**Figure 1:**
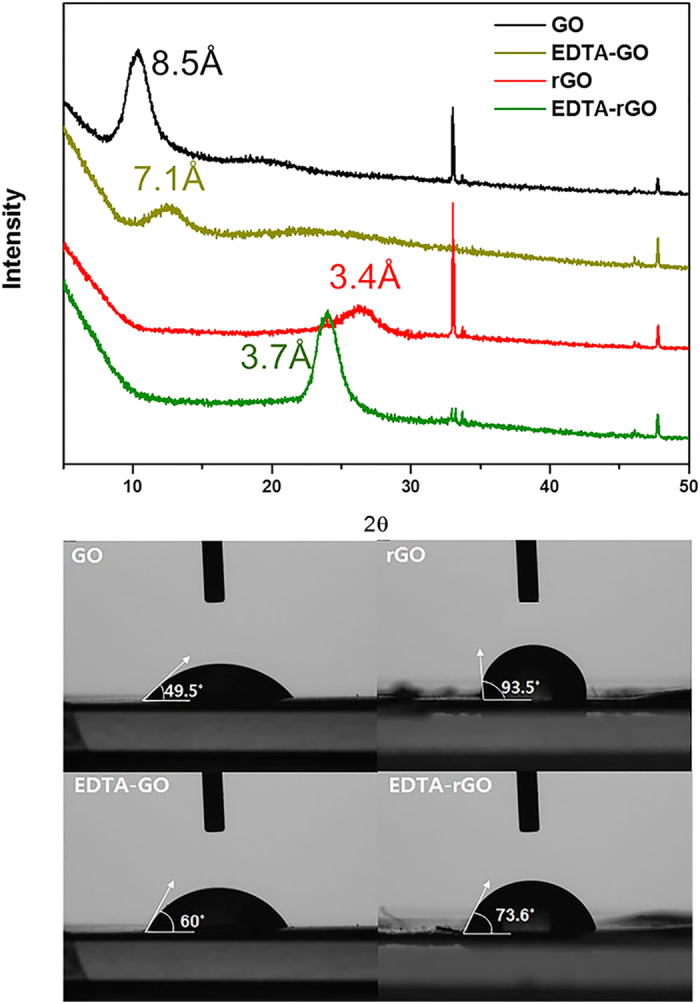
Characterization of EDTA-graphene. (**a**) X-ray diffraction for GO, EDTA-GO, rGO and EDTA-rGO. (**b**) Contact angles of water on GO, rGO, EDTA-GO and EDTA-rGO membrane.

The buckypapers of GO, rGO, EDTA-GO, and EDTA-rGO were removed from the PTFE membrane sheet and analyzed for the interlayer spacing. Figure 1a shows the interlayer spacing as determined by X-ray diffraction (XRD). Immediately notable in the figure is the substantial reduction in the interlayer spacing as GO and EDTA-GO nanoplates were reduced to prepare rGO and EDTA-rGO membranes. The chemical reduction of GO with hydrazine resulted in the recovery of π-π conjugation within the graphene^22^,^23^,^24^ and the interlayer spacing decreased to 3.4 Å (rGO) from 8.5 Å as GO was reduced to rGO. The spacing decreased to 3.7 Å from 7.1 Å as EDTA-GO was reduced to EDTA-rGO. Another notable finding here is that the spacing decreased to 7.1 Å from 8.5 Å when GO was functionalized to EDTA-GO whereas it increased to 3.7 Å from 3.4 Å when rGO was functionalized to EDTA-rGO. As indicated earlier, the substantial decrease in the spacing following the chemical reduction can be attributed to the removal of functional groups on GO. The increase in the spacing after the functionalization of rGO to EDTA-rGO is due to the presence of the EDTA functional group that rGO does not have.

The decrease in the spacing after the functionalization of GO to EDTA-GO, however, involves more factors than in the case of functionalized rGO. Replacing the hydroxyl group, where the EDTA group forms, with the EDTA group should result in a slight increase in the spacing because of the larger size of EDTA compared to the hydroxyl group. The functionalization, however, must have led to partial reduction of other functional groups, resulting in a decrease in the spacing. To functionalize GO with EDTA, GO was reacted with EDTA-silane at 60–65 °C for 12 h in water. Although the temperature is not quite high, hydrothermal reduction should occur. This reduction can be verified from the contact angles shown in Fig. 1b for four types of buckypapers. As shown, the contact angle increases to 60° from 49.5° when GO was functionalized to EDTA-GO, indicating a shift toward hydrophobicity, which is a result of partial chemical reduction in the course of functionalization. The partial chemical reduction is demonstrated from the decrease in the peak intensity of C=O (1733 cm^−1^) and an epoxy group (1174 cm^−1^), as observed from the FT-IR spectra in Supplementary Fig. 4. Figure 1b also shows that when GO is chemically reduced to rGO, the water contact angle increases to 94°, making the membrane hydrophobic. This hydrophobicity is the main reason why rGO membrane is practically impermeable to water^19^. Because of the addition of hydrophilic functional group of EDTA to rGO, the contact angle of EDTA-rGO is lower than that of rGO. EDTA-rGO membrane becomes somewhat hydrophilic as indicated by the contact angle of 74°. Because of this hydrophilicity, EDTA-rGO membrane is permeable to water. The existence of the EDTA on the rGO plates after chemical reduction can be confirmed in Supplementary Fig. 5. Supplementary Fig. 6 shows the surface and cross-sectional scanning electron microscopy (SEM) images of EDTA-GO and EDTA-rGO layer on PTFE membrane. When a 3 ml volume of the solutions (concentration: 1 mg/ml) is filtered, the thickness of the layer becomes approximately 1 μm for EDTA-GO and 0.9 μm for EDTA-rGO membrane. As shown in Supplementary Fig. 6a,b, the surface of the EDTA-GO is rougher than that of EDTA-rGO. The rough surface may be due to a difference in dispersibility characteristics in water. Over a period of time, some precipitates of EDTA-GO were observed to form in water whereas EDTA-rGO was stable. These precipitates can be aggregates of EDTA-GO sheets that are bonded together by bonding between the Na^+^ ion at the end of EDTA molecules on EDTA-GO nanosheets and the oxygen functional groups on other sheets. The peak (1675 cm^−1^) of Na-O-C related to Na^+^ ion and oxygen bonding is observed in FTIR spectra of EDTA-GO^25^ but the bonding peak is not observed in that of EDTA-rGO. It is known that a rough (Wensel) surface is more water-wettable than a smooth surface^26^. Therefore, the smaller contact angle of EDTA-GO (60°), compared to that of EDTA-rGO (73.6°), would be due to the rough surface of EDTA-GO. No significant defects and cracks were observed on both membranes. The cross-sectional SEM images in Supplementary Fig. 6c,d show that the platelets are well stacked.

## Ion Sieving of EDTA-Graphene Membranes

To demonstrate the utility of the functionalized GO and rGO membranes for ion sieving, the ion permeation rates through the membranes were measured with a U-shaped tube^18^ shown in Supplementary Fig. 7. The feed and permeate compartments of the U-tube were initially filled to the same height with an ion solution such as KCl, NaCl, MgCl_2_ and K_3_Fe(CN)_6_ in the feed side and deionized water (DI water) in the permeate side. The graphene membranes were tightly sealed with silicone rubber gaskets to prevent leakage of feed and permeate solutions. Diffusion of ions and water molecules is induced by a concentration difference between the ion and DI solutions. Therefore, osmotic pressure is the main driving force for the transport of ions and water molecules in the measurement tube. The osmotic pressure is ≈49 and 0.5 bar for 1 molar (M) and 10 mM NaCl solution, respectively, at room temperature because van’t Hoff factor is 2 for NaCl (Supplementary Fig. 8 inset). There are no external forces. The hydrostatic pressure in our experiments does not exceed ≈10^−2^ bar. For 1 μm thick EDTA-GO membrane and 0.9 μm thick EDTA-rGO membrane, the water permeation rates were approximately 0.8 l/m^2^/h (LMH) and 0.4 LMH, respectively, which is the rate of water flow from the permeate compartment (pure water) to the feed compartment (ion solution)) when NaCl concentration was 1 M in the feed. The permeability of EDTA-GO was higher than that of EDTA-rGO due to the difference in hydrophilicity and interlayer spacing. As shown in Supplementary Fig. 8, the permeability increased with increasing molar concentration because of increasing osmotic pressure with increasing feed concentration.

**Figure 2:**
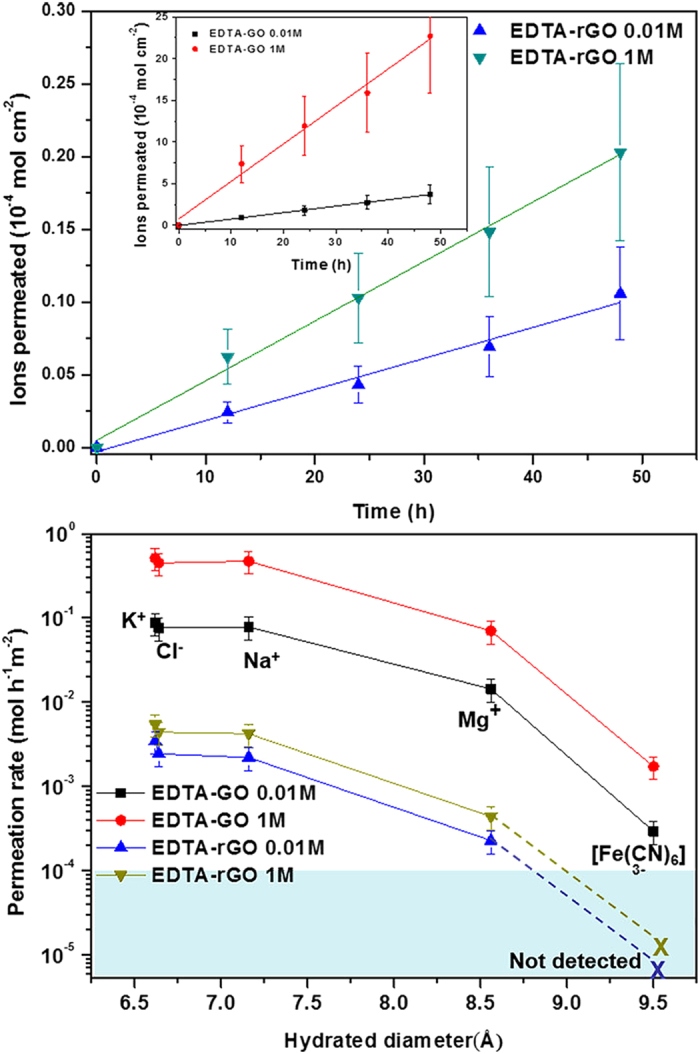
Ion permeation through EDTA-GO and EDTA-rGO membranes. (**a**) Ions permeated through a 0.9 μm-thick EDTA-rGO membrane from 1 M and 0.01 M NaCl solution. (Inset) Ions permeated through a 1 μm-thick EDTA-GO membrane from 1 M and 0.01 M NaCl solution. (**b**) Ion permeation rates of various ions through EDTA-GO and EDTA-rGO membranes at two different concentrations of 0.01 and 1 M. No permeation of [Fe(CN)_6_]^3−^ through EDTA-rGO could be detected within light blue area during measurements lasting for 7 days. Standard deviation of data obtained in this work is within 30%.

Figure 2a shows the amount of ions permeated through the graphene membranes as a function of time for two concentrations of NaCl in water. The ion concentration of the permeate solution was measured by inductively coupled plasma(ICP) and ion chromatography(IC) after the ion permeation tests for 48 hrs. The amount of ions permeated or the ion concentration in the permeate compartment increased linearly with time for both types of membranes whether the NaCl concentration is 1 M or 0.01 M in the feed compartment. The slopes of these lines are the permeation rates. The amount of permeated ions increased with increasing concentration due to the corresponding increase in the driving force. The figure shows that the permeation rate through the EDTA-GO membrane (Fig. 2a, inset) is higher than that through the EDTA-rGO membrane for the same feed concentration. A larger interlayer spacing of EDTA-GO than that of EDTA-rGO along with more hydrophilic nature of the GO membrane is responsible for the larger permeation rate.

The permeation rates determined for five ions are shown in Fig. 2b. As one might expect, the ion permeation rate is higher for smaller ion or smaller hydrated diameter. The rate for Cl^−^ was measured with NaCl. Cations and anions move through membranes in stoichiometric amounts so that the charge neutrality within each of the compartment is preserved. Cl^−^ is similar to Na^+^ in the permeation rate. EDTA-rGO shows no detectable permeation of large sized [Fe(CN)_6_]^3−^. The permeation rates for small ions of potassium and sodium through the EDTA-rGO membrane, which are 5.5 × 10^−3^ for K^+^ and 4.2 × 10^−3^ for Na^+^, respectively, are three orders of magnitude lower compared to those through GO membrane reported earlier^18^. These very low ion permeation rates through EDTA-rGO membrane resulted from a narrow interlayer spacing between the EDTA-rGO nanoplates in wetted state, which is smaller than the hydrated diameters of the small ions.

**Figure 3:**
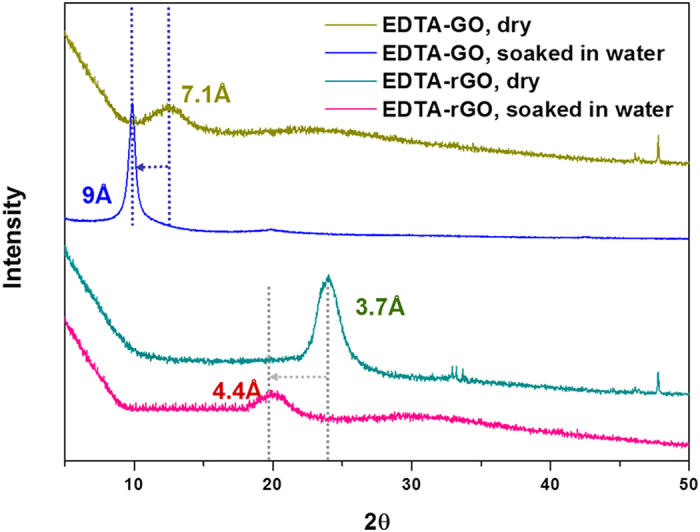
X-ray diffraction results for dry and wetted EDTA-GO and EDTA-rGO.

To help explain the very low permeation rates through the EDTA-rGO membrane, the interlayer spacing of EDTA-graphene membranes was measured by XRD. Figure 3 shows the interlayer spacing in dry and wetted state. While the interlayer spacing increased to 9.0 Å from 7.1 Å when wetted for EDTA-GO membrane, the spacing increased only to 4.4 Å from 3.7 Å for EDTA-rGO membrane, a mere 0.7 Å increase when the dry membrane was wetted. The interlayer spacing of 4.4 Å for the wetted EDTA-rGO is much smaller than the hydrated diameters of the small ions, which are 6.62 Å for K^+^ and 7.16 Å for Na^+ ^27,28,29,30, leading to a three orders of magnitude decrease in the ion permeation rates compared to the permeation rates through the usual GO membrane^18^. Without any functionalization, the swelling of GO membrane due to intercalated water was significant, the interlayer spacing increasing to 13 Å from 9 Å upon wetting^18^. The reduction in swelling when GO is functionalized has to do with an increase in the hydrophobicity that accompanies the EDTA functionalization. Hydrophobicity tends to limit intercalation of water into the interlayer space. For instance, hydrophobic film containing rGO nanoplates makes the film impermeable to water^19^. As shown in Fig. 1b, the water contact angle increases from 49.5° to 60° as the GO is functionalized to EDTA-GO, making it more hydrophobic. This more hydrophobic nature of EDTA-GO resulted in a reduction in swelling compared to GO membrane. When rGO is functionalized to EDTA-rGO, the figure shows that the contact angle becomes larger than that for EDTA-GO, which would lead to a further reduction in swelling and thus a reduction in the interlayer spacing.

To investigate the relationship between interlayer spacing and ion permeation, we determined the permeation rate of Na and Mg ions as a function of the interlayer spacing (Supplementary Fig. 9). The results show that the ion permeation rate of both ions declines with decreasing interlayer spacing. In particular, a significant decline in the rate results when the interlayer spacing is smaller than the diameter of the hydrated ions (blue vertical dotted line for Na and red for Mg). This trend is similar to that observed in the conventional membranes, which is attributed to size exclusion mechanism. The data for the interlayer spacing of 13 Å are from Joshi *et al*.^18^ for a GO membrane.

The angstrom-level membrane with an interlayer spacing of 3.7 Å bodes well for its utility in the areas of gas separation, and genetic and medical diagnostics, as demonstrated here for ion sieving. An inherent problem of swelling due to an intercalated water or fluid can be minimized, if the results shown here for water are an indication, by modifying and adjusting the functionality so as to make it more hydrophobic or oleophobic depending on the type of fluid. Such efforts would lead to a GO-based membrane with an interlayer spacing of 3.4 Å that is little affected by an intercalated fluid.

## Materials and Methods

### Preparation of EDTA functionalized graphene membrane

GO was prepared from graphite powders according to Hummers’ method. To functionalize GO, 50 ml of 1 mg/ml GO aqueous solution was prepared. Then 5 ml of 1.0% aqueous solution of N-(trimethoxysilylpropyl) ethylenediamine triacetic acid(EDTA-silane) was added and stirred for 12 h at 60–65 °C for silylation^20^. After the reaction was finished, 100 ml of methanol was added to dilute the unreacted silane molecules. EDTA-GO was acquired by filtration (PTFE filter: Millipore JHWP 04700 pore size: 450 nm) and washed with methanol and water sequentially. After the filtration and washing is completed, EDTA-GO was dried in vacuum oven at 70 °C for 48 h.

To reduce EDTA-GO, the dried EDTA-GO was redispersed in DI water and reduced with hydrazine. Hydrazine was added into the EDTA-GO solution (1 μl hydrazine per 5 mg GO) and then stirred for at least 8 h at 80 °C. After the reduction, the product EDTA-rGO was obtained by filtration, then over-dried for further use.

### Ion Permeation Test

EDTA-GO and EDTA-rGO buckypapers were prepared by filtering the dispersions and then vacuum drying on PTFE membrane (pore size: 450 nm, Millipore JHWP 04700) as a support layer. When 3 ml volume of the solutions was filtered, the thickness was approximately 1 μm for EDTA-GO and 0.9 μm for EDTA-rGO. The buckypaper membranes were placed between two L-shaped glass tubes(refer to supplementary Fig 7). Silicon rubber gaskets were put over both sides of the membrane to prevent solution leakage.

The side of graphene buckypaper in the membrane was set toward ion solution. Two L-shaped glass tubes in which the membrane was set were tightly clamped with metal clamp. After the test cell was placed on magnetic stirrer, ion solution and DI water were allowed to fill to the same level in both sides of the cell, respectively. The ion permeation was measured for 48 hours under vigorous stirring to prevent concentration polarization that can occur near the membrane. The same amount of ion solution and DI water was collected every 12 hours to measure the ion permeated according to the measurement time. Ion concentration of collected solutions was measured thorough Inductively Coupled Plasma (ICP) and Ion Chromatograph (IC).

### Characterization

The microstructures of the hybrid structures based on graphene sheets are characterized by scanning electron microscope (SEM) images and EDS spectra, taken with a Carl Zeiss SUPRA 55VP FE-SEM. For revealing the compositions of the hybrid structures, X-ray diffraction(XRD) analysis was performed on a Bruker New D8-advance X-ray diffractometer equipped with Ge-monochromatized Cu Kα radiation (λ = 1.5418 Å). For Fourier transform infrared spectrometry (Thermo Scientific Nicolet 670), GO, EDTA-GO and EDTA-rGO were scanned from 500 to 4000 cm^−1^ in forms of buckypaper. Contact angle of GO, rGO, EDTA-GO and EDTA-rGO layer on PTFE filter was measured with KRÜSS DSA 100 instrument. Conductivity of ion solutions (MgCl_2_, NaCl and KCl solutions) before and after filtration for permeation rate was measured with Mettler Toledo SevenCompact^TM^ conductivity meter. The concentration of positive and negative ions into the solution was measured with Shimdzu JP/ICPS-750 (inductively coupled plasma, ICP) and Dionex ICS-3000(Ion chromatograph, IC), respectively.

## Additional Information

**How to cite this article**: Lee, B. *et al*. Membrane of Functionalized Reduced Graphene Oxide Nanoplates with Angstrom-Level Channels. *Sci. Rep*. **6**, 28052; doi: 10.1038/srep28052 (2016).

## Supplementary Material

Supplementary Information
