# Supplementary material for: Membrane of Functionalized Reduced Graphene Oxide Nanoplates with Angstrom-Level Channels
Source: Sci Rep. 2016 Jun 16;6:28052. doi: 10.1038/srep28052 (PMC4910041; doi:10.1038/srep28052)
Supplement: Supplementary Information [file srep28052-s1.pdf]

Membrane of Functionalized Reduced Graphene Oxide Nanoplates with Angstrom-Level Channels

Byeongho Lee[1,2], Kunzhou Li[2], Hong Sik Yoon[3], Jeyong Yoon[3,4], Yeongbong Mok[5], Yan Lee[5], Hong H. Lee[3*] and Yong Hyup Kim[2*]

[1]*BK21 Plus Program in Chemical Engineering, Seoul National University, Daehak-dong, Gwanak-gu, Seoul 151-742, Republic of Korea.*

[2]*School of Mechanical and Aerospace Engineering, Seoul National University, Daehak-dong, Gwanak-gu, Seoul 151-742, Republic of Korea*

[3]*School of Chemical and Biological Engineering, College of Engineering, Institute of Chemical Processes(ICP), Seoul National University(SNU), Gwanak-gu, Daehak-dong, , Seoul 151-742, Republic of Korea.*

[4]*Asian Institute for Energy, Environment & Sustainability (AIEES), Seoul National University (SNU), Gwanak-gu, Daehak-dong, Seoul 151-742, Republic of Korea*

[5]*Department of Chemistry, Seoul National University, Daehak-dong, Gwanak-gu, Seoul 151-747, Republic of Korea*

*To whom correspondence should be addressed. E-mail: yongkim@snu.ac.kr (Y. K.); honghlee@snu.ac.kr (H. L.)

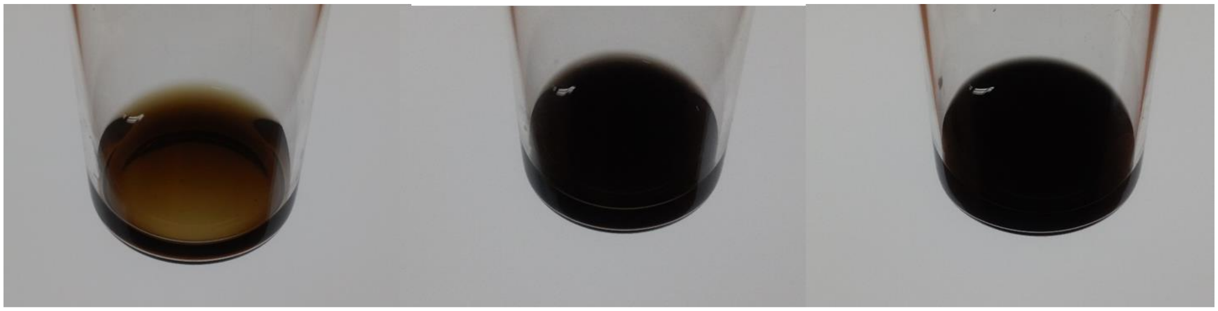

Supplementary Figure 1 | Photographs of GO, EDTA-GO and EDTA-rGO solutions. All the solutions are 1 mg/ml in concentration and 3 ml in volume. GO solution is semi-transparent and brown but EDTA-GO and EDTA-rGO are opaque and black.

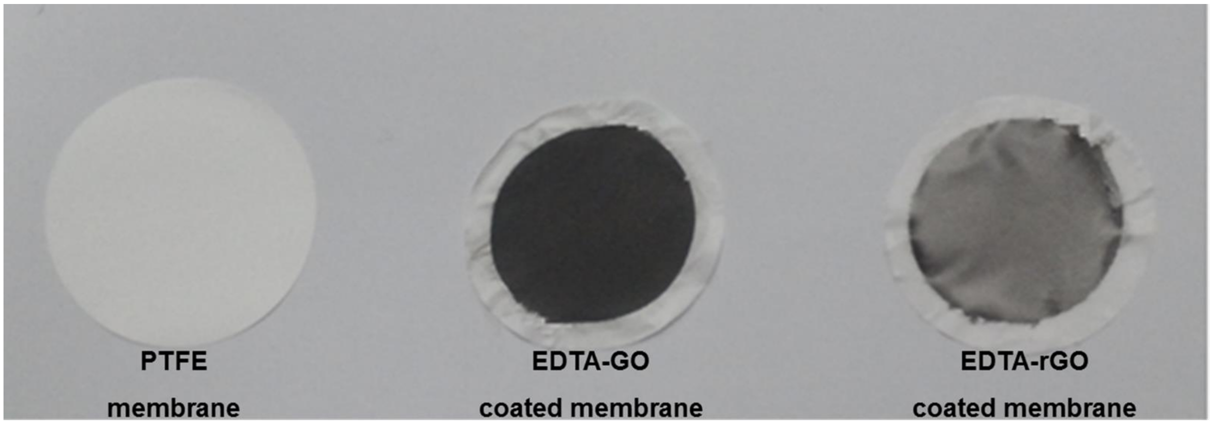

Supplementary Figure 2 │ PTFE membrane used as support layer (left, pore size: 0.45 µm, membrane diameter: 47 mm), EDTA-GO (middle), and EDTA-rGO (right) on the PTFE. 3ml was filtrated.

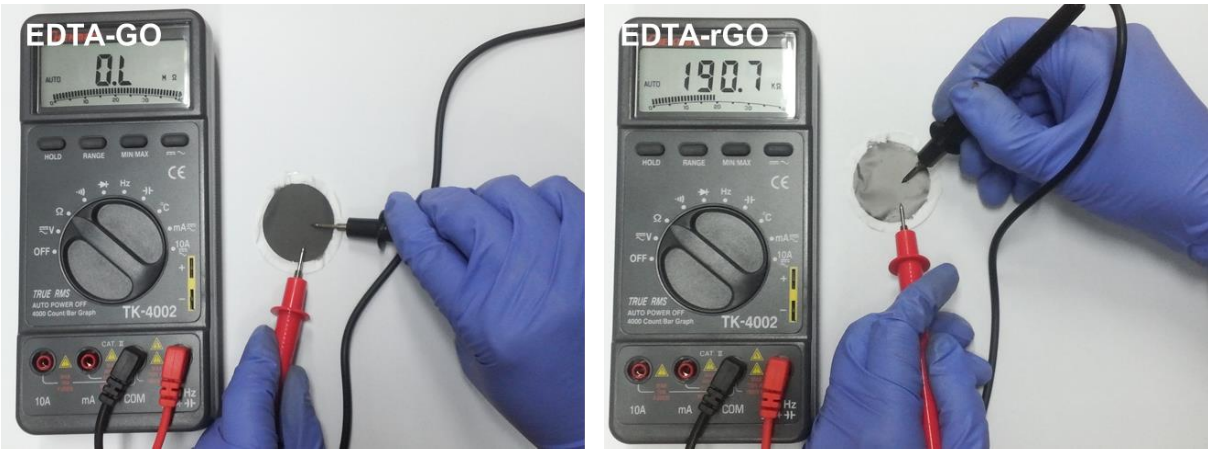

Supplementary Figure 3 | Multi-meter test for electrical characteristics of EDTA-GO and EDTA-rGO on PTFE filter. EDTA-GO is non-conductive but EDTA-rGO is conductive

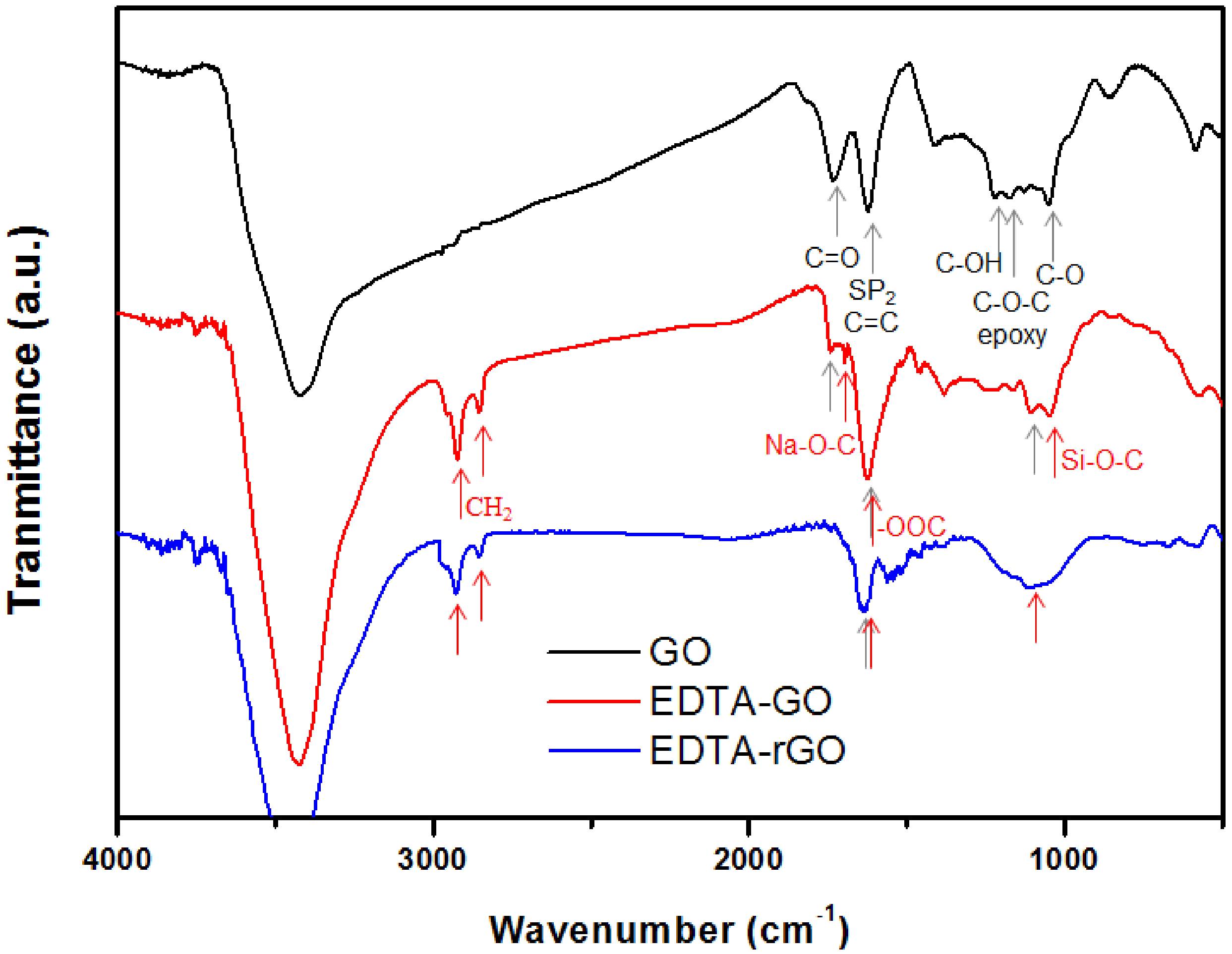

Supplementary Figure 4 │ FT-IR spectra of GO, EDTA-GO and EDTA-rGO. Gray arrows are for GO peaks and the red ones are for EDTA peaks and bonding peaks between EDTA and GO.

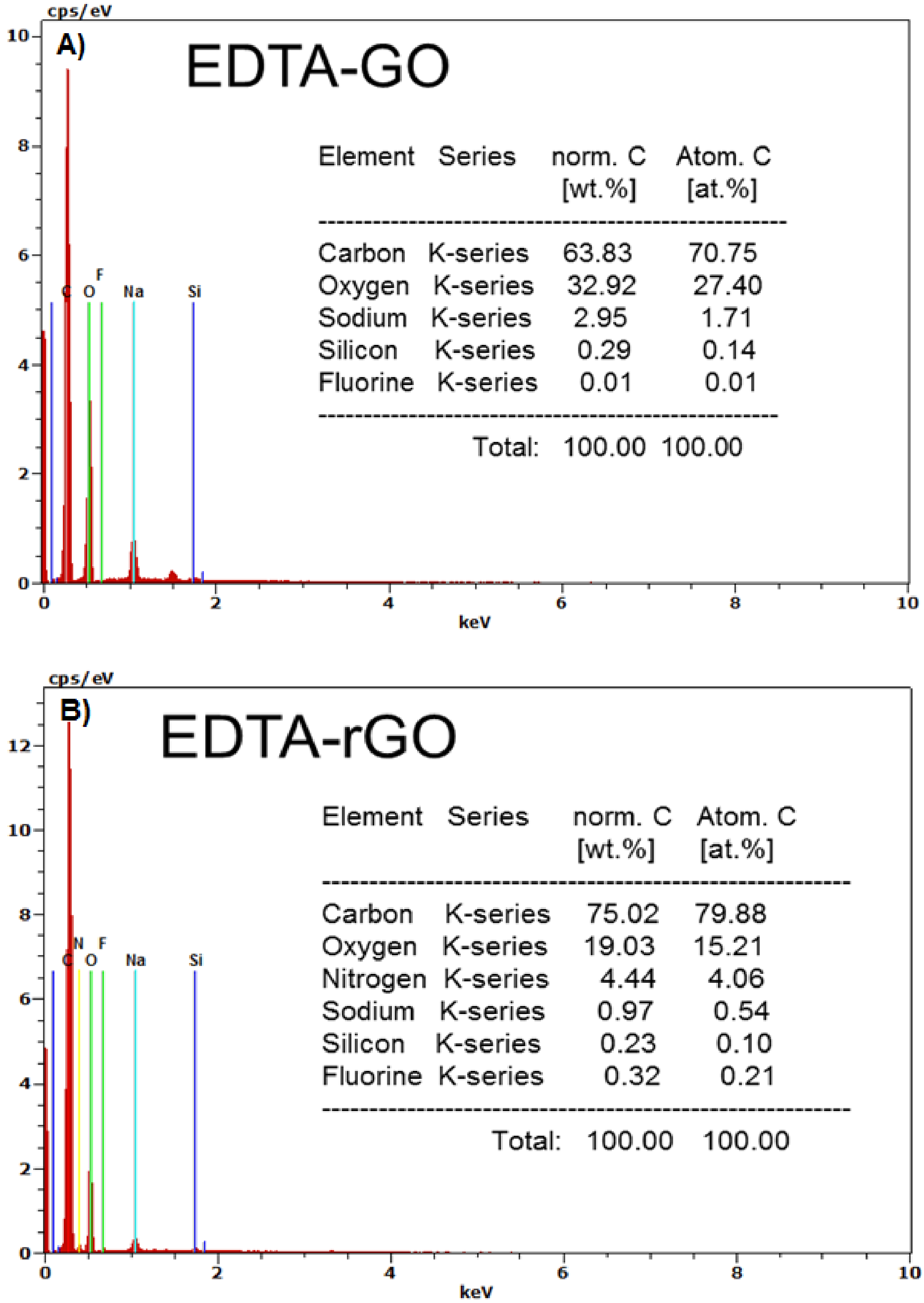

Supplementary Figure 5 | EDS spectra of (A) EDTA-GO and (B) EDTA-rGO on PTFE filter. Sodium and silicon peaks from EDTA are observed. Fluorine peak comes from PTFE filter.

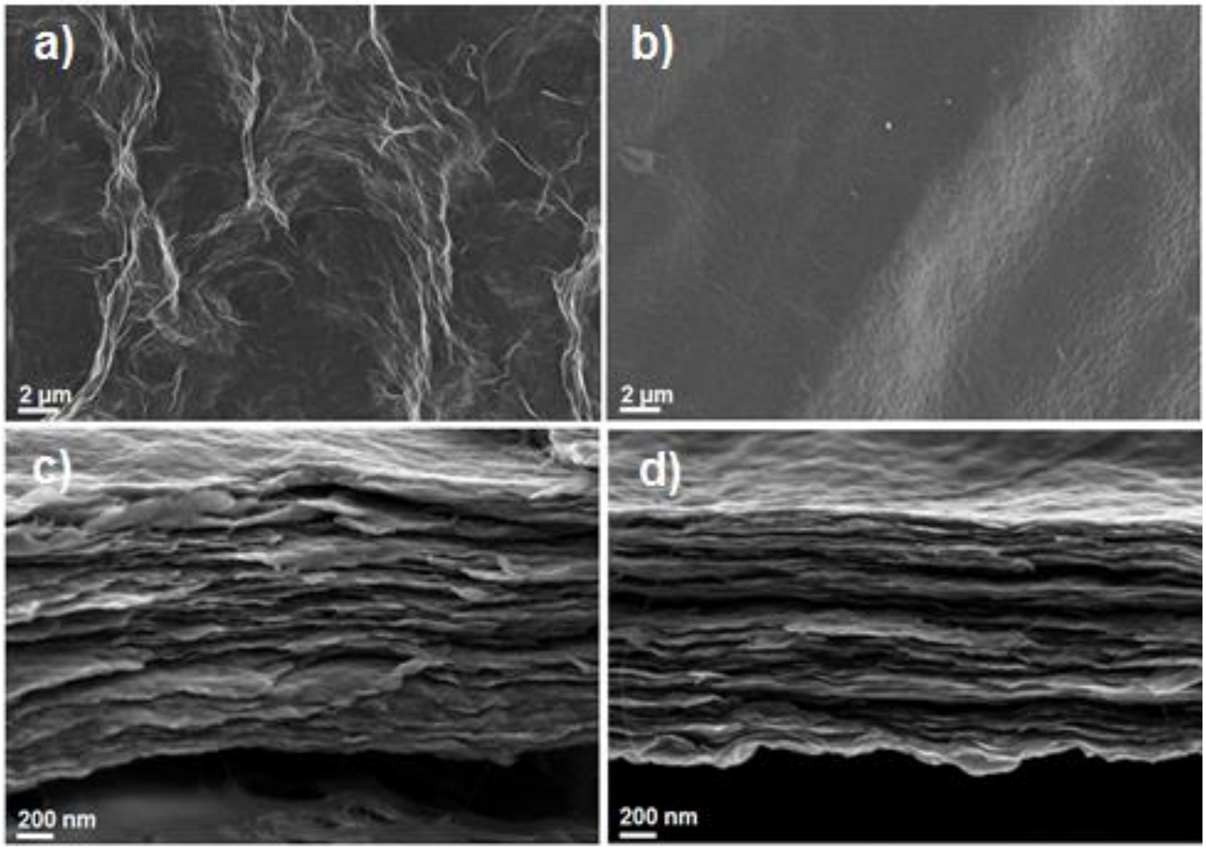

Supplementary Figure 6 | Surface ((a) and (b)) and cross-section ((c) and (d)) SEM images of EDTA-GO (left) and EDTA-rGO membrane (right) on PTFE filter (support).

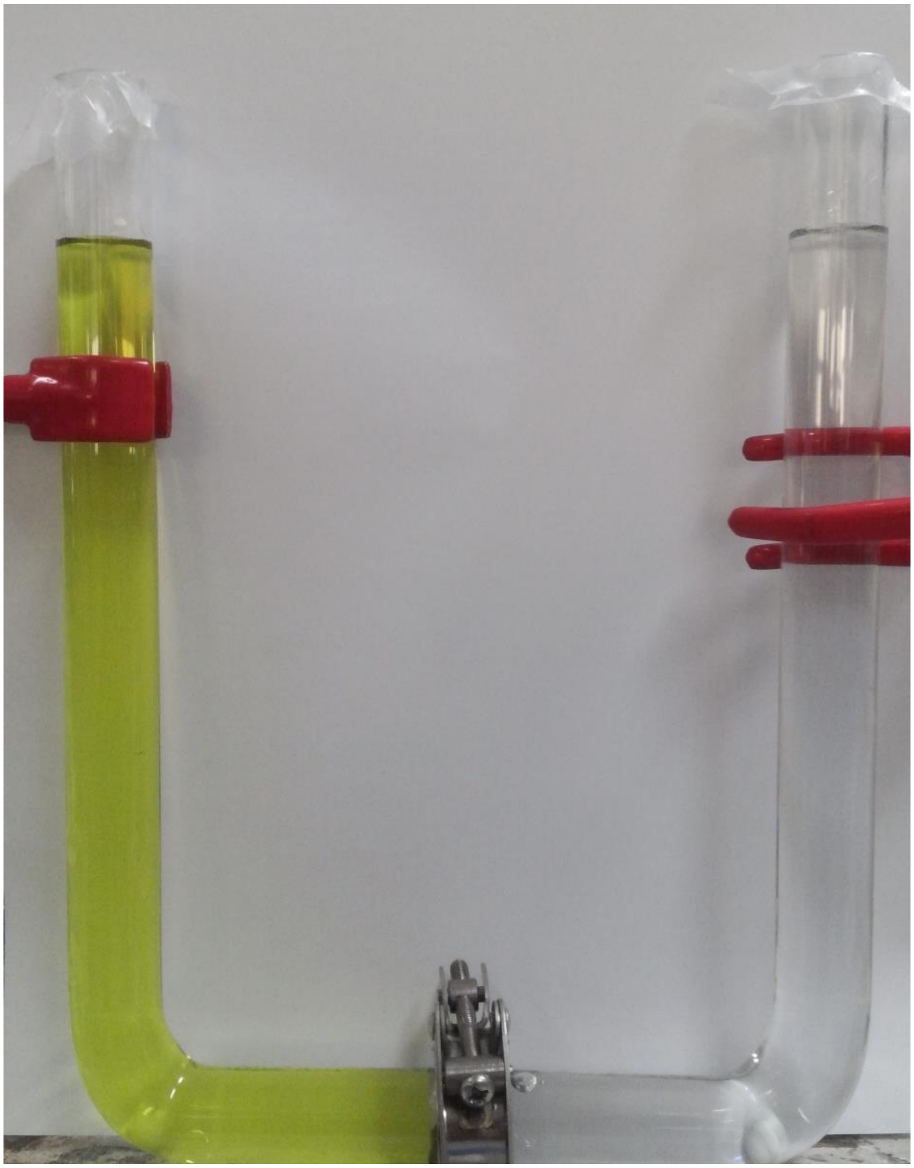

Supplementary Figure 7 │ Photograph of experimental setup. U-shaped test cell for ion permeation experiments. 1 M aqueous solution of $K_3Fe(CN)_6$ in feed compartment(left in the above image, green colored solution) and DI water in permeate (right in the above image). Ion permeation was tested for 48 hours without external pressure.

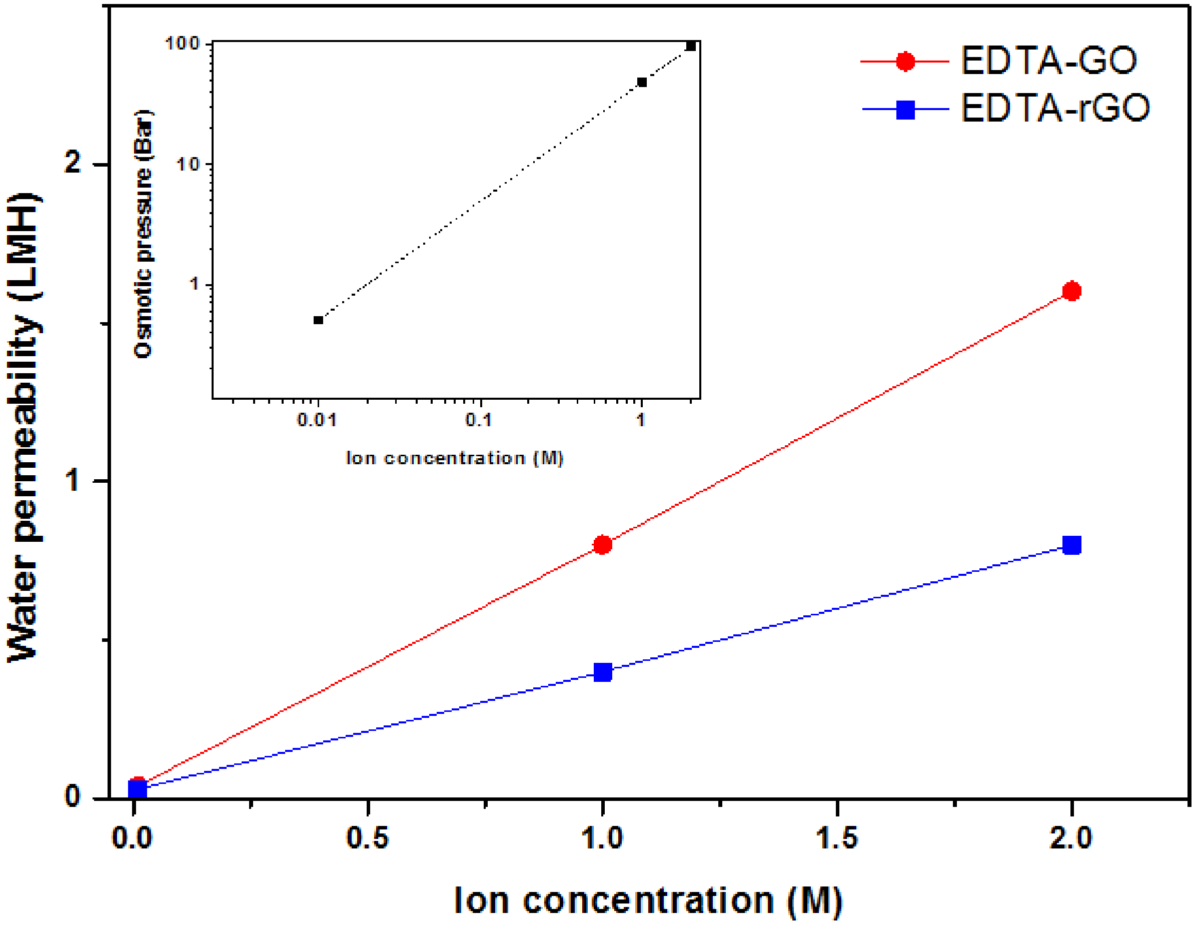

Supplementary Figure 8 │ Water permeability as a function of ion concentration. The permeability through EDTA-GO(red) and –rGO(blue) was measured with NaCl. Inset: calculated osmotic pressure as a function of ion concentration. The van't Hoff factor is 2 for NaCl.

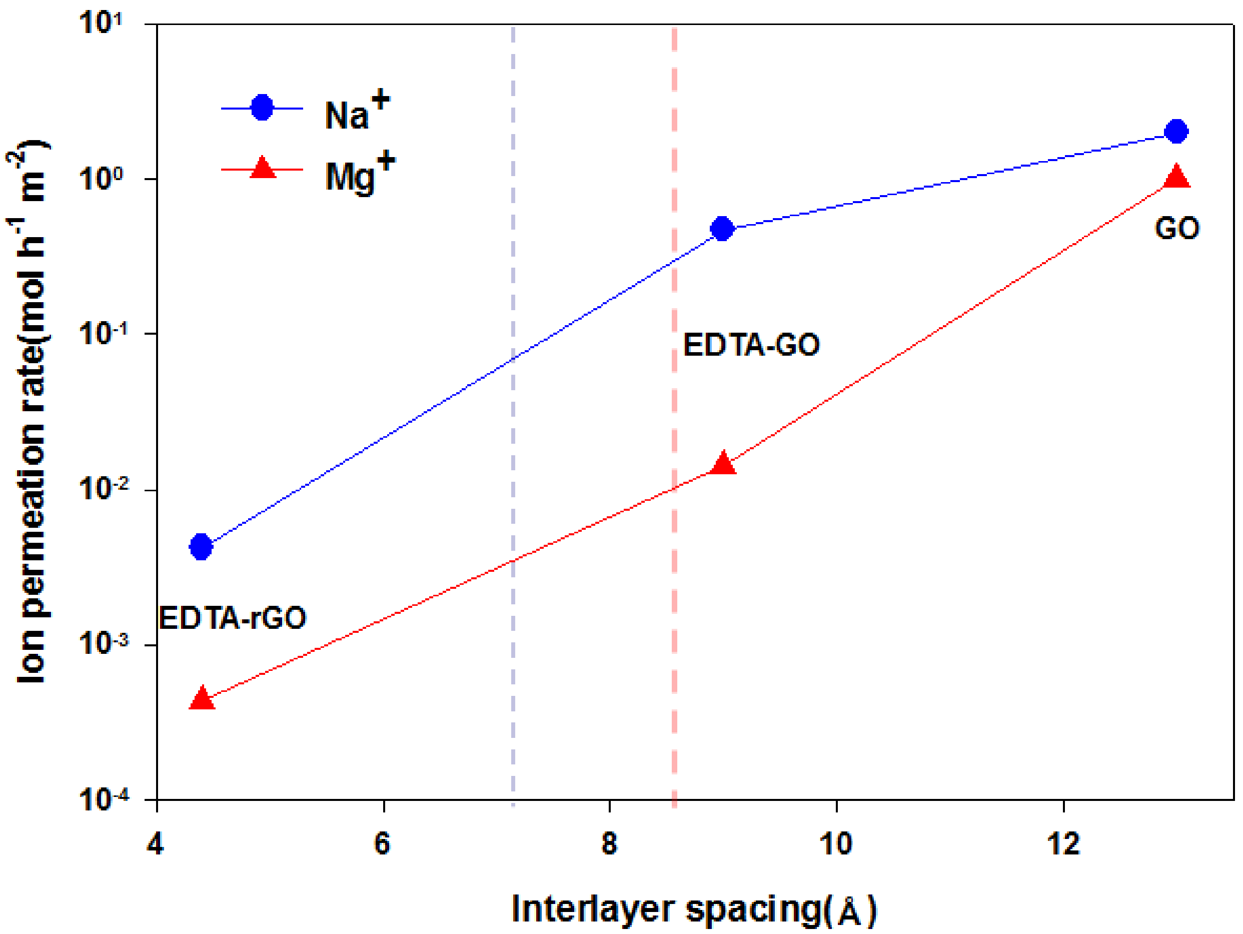

Supplementary Figure 9 │ Relationship between ion permeation and interlayer spacing. Vertical blue and red dashed lines show the sizes of hydrated Na and Mg ion. Note that the data for the interlayer spacing 13Å are from Joshi et al[1] in wetted state.

Reference

1. Joshi R, Carbone P, Wang F, Kravets V, Su Y, Grigorieva I, *et al.* Precise and ultrafast molecular sieving through graphene oxide membranes. *Science* 2014, **343**(6172)**:** 752-754.
